# Development and Characterization of a *Nanophyton iliense*-Based Gel for Topical Application

**DOI:** 10.3390/pharmaceutics18060710

**Published:** 2026-06-09

**Authors:** Lashyn N. Kiyekbayeva, Serzhan E. Mombekov, Moldir K. Kudaibergenova, Nursulu Z. Akhtayeva, Assem T. Mamurova, Ayala S. Mukhametzhan, Yelzhas Nurlykhan, Rizvangul B. Ayupova, Galiya S. Ibadullayeva, Yelena V. Sitdikova, Gulnaz N. Musina

**Affiliations:** 1 School of Pharmacy, Kazakh National Medical University Named After S.D. Asfendiyarov, St. Tole Bi 94, Almaty 050000, Kazakhstan; lashynk@mail.ru (L.N.K.); mse_09.09.91@mail.ru (S.E.M.); manali89@mail.ru (M.K.K.); nurlyhanelzhas@gmail.com (Y.N.); 2 Department of Biodiversity and Bioresources, Faculty of Biology and Biotechnology, Al-Farabi Kazakh National University, Almaty 050040, Kazakhstan; akhtaeva@mail.ru (N.Z.A.); amamurova81@mail.ru (A.T.M.); 3 International School of Medicine, Kazakh National Medical University Named After S.D. Asfendiyarov, St. Tole Bi 94, Almaty 050000, Kazakhstan; amukhametzhan2007@gmail.com; 4 Department of Chemistry and Chemical Technology, Karaganda Technical University Named After Abylkas Saginov, Karaganda 100000, Kazakhstan; lena.alive@mail.ru (Y.V.S.); gulnaz_musina@mail.ru (G.N.M.)

**Keywords:** *Nanophyton iliense*, polyphenols, antioxidant activity, DPPH assay, gel formulation, topical delivery, phytochemicals

## Abstract

**Background:** *Nanophyton iliense* U.P. Pratov is a Central Asian halophytic plant whose phytochemical composition and suitability for pharmaceutical formulation remain insufficiently explored. This study evaluated the chemical profile, antioxidant activity, preliminary safety, and gel-forming potential of a hydroethanolic extract of *N. iliense* for topical application. **Methods:** The extract was characterized by GC–MS and HPLC. Total polyphenol content was determined, antioxidant activity was assessed using FRAP and DPPH assays, and preliminary cytotoxicity was evaluated using the Artemia salina lethality assay. **Results:** GC–MS and HPLC analyses showed that the extract contained both lipophilic constituents, including terpenoid and phytosterol-related compounds, and phenolic compounds such as catechin, epicatechin, and naringin. The total polyphenol content reached 485.05 mg GAE/L, exceeding the values obtained for the other plant extracts analyzed under the same conditions. The extract showed concentration-dependent antioxidant activity in both FRAP and DPPH assays. In the DPPH assay, radical scavenging activity increased up to 90.06% at 1.0 mg/mL, while FRAP results confirmed a strong reducing capacity. In the *Artemia salina* assay, no cytotoxic effect was observed at the tested concentrations. To assess pharmaceutical applicability, five gel formulations were prepared and compared. Gel No. 4, containing *N. iliense* extract, Lecigel, glycerin, Tween 80, benzyl alcohol, and purified water, showed the most suitable organoleptic and technological characteristics, including homogeneity, good spreadability, and absence of greasy residue. **Conclusions:** The obtained results indicate that *N. iliense* extract can be incorporated into a semi-solid formulation, while the extract itself demonstrates relevant in vitro antioxidant properties.

## 1. Introduction

Halophytic plants are adapted to saline and arid environments, where they are exposed to intense abiotic stress. These conditions can stimulate the accumulation of secondary metabolites, including phenolic compounds, flavonoids, terpenoids, and sterol-related constituents, which may contribute to antioxidant and protective activity [1,2,3]. *Nanophyton iliense* U.P. Pratov is a poorly studied halophytic species growing in Kazakhstan. Although previous phytochemical data suggest that this plant may contain biologically active metabolites [4], its potential as a source of extract-based topical formulations has not been sufficiently evaluated. Therefore, the development of a suitable semi-solid delivery system may help assess the practical pharmaceutical relevance of this species [5,6,7].

The added value of the present study lies in connecting the previously reported phytochemical potential of *N. iliense* with its practical formulation development. In particular, it has not been clearly demonstrated whether a chemically complex extract containing both polar phenolic compounds and lipophilic constituents can be incorporated into a homogeneous semi-solid formulation for topical use. Thus, this work focuses not only on the evaluation of extract composition and antioxidant activity, but also on its translation into an initial gel formulation followed by preliminary pharmaceutical quality assessment.

Plant-derived secondary metabolites, including phenolic compounds, flavonoids, and terpenoids, have attracted significant attention due to their diverse biological and therapeutic potential, including antioxidant, protective, and disease-modulating effects [8]. Oxidative stress, caused by an imbalance between the production of reactive oxygen species (ROS) and the antioxidant defense system, is known to play a key role in the pathogenesis of numerous diseases, including chronic inflammation, cardiovascular disorders, and neurodegenerative conditions [9]. Therefore, the search for new natural antioxidants remains an important direction in modern pharmaceutical research.

Among plant metabolites, phenolic compounds are of particular interest due to their ability to act as free radical scavengers, metal chelators, and reducing agents [10]. These properties contribute to their capacity to inhibit oxidative processes and protect biological systems from damage. However, despite their promising biological activity, the practical application of phenolic-rich plant extracts is often limited by several factors, including chemical instability, low bioavailability, and insufficient penetration into target tissues [11,12].

To address these limitations, increasing attention has been devoted to the development of drug delivery systems that can enhance the stability and effectiveness of bioactive compounds. Topical formulations, especially gel-based systems, represent a convenient and efficient approach for the localized administration of active substances. Gels are characterized by favorable rheological properties, ease of application, and the ability to form uniform films on the skin surface, which can improve the retention and controlled release of active compounds. In addition, polymer-based gels, such as those prepared using carbopol or carboxymethylcellulose, are widely used in pharmaceutical practice due to their biocompatibility, stability, and ability to modulate drug release [13,14].

*N. iliense* U.P. Pratov is a halophytic plant species native to arid and saline regions of Central Asia. Plants growing under such extreme environmental conditions are known to accumulate a diverse range of secondary metabolites as part of their adaptive mechanisms [15,16,17]. These compounds, including phenolics, flavonoids, and triterpenoids, are often associated with pronounced biological activities [18,19]. Despite its ecological significance and potential as a source of bioactive substances, the integration of *N. iliense* bioactive compounds into pharmaceutical formulations remains insufficiently studied.

The aim of this study was to characterize the phytochemical composition of *N. iliense* extract, evaluate its antioxidant and preliminary safety profile, and develop a gel formulation suitable for preliminary topical formulation studies. The study combines botanical, chemical, biological, and technological evaluation in order to determine whether the extract can be incorporated into a stable semi-solid dosage form.

## 2. Materials and Methods

### 2.1. Plant Material

The aerial parts of *N. iliense* U.P. Pratov were collected in 2024 during the flowering stage in the foothills of the Bugaty region, Kazakhstan. The plant material was identified using classical botanical methods, including route-orientational, ecological-systematic, and ecological-geographical approaches. Taxonomic identification was performed according to the reference work Flora of Kazakhstan and verified using international botanical databases including the International Plant Names Index (IPNI) and Plants of the World Online (POWO).

The collected plant material was cleaned from soil impurities, washed with distilled water, and fixed in 40% ethanol prior to anatomical analysis.

### 2.2. Morphological and Anatomical Analysis

Morphological characteristics of *N. iliense* were studied using standard botanical methods. Diagnostic structures of the plant were photographed using a digital camera attached to a microscope. For anatomical analysis, transverse sections of the plant stem were prepared from fixed plant material using an OL-ZCO 30 freezing microtome (INMEDPROM, Yaroslavl, Russia) according to the generally accepted pharmacognostic method described in the State Pharmacopoeia of the Republic of Kazakhstan [20].

The prepared sections were mounted on slides, covered with coverslips, and examined using an MC-300 microscope (Micros Handelsgesellschaft m.b.H., Vienna, Austria) at ×180 magnification. Microphotographs were obtained for documentation and morphometric analysis.

### 2.3. GC–MS Analysis

Chemical profiling of the plant extracts was performed using gas chromatography–mass spectrometry (GC–MS). The analysis was carried out on an Agilent 7890A gas chromatograph equipped with an Agilent 7693 autosampler and coupled with an Agilent 5975C mass spectrometer (Agilent Technologies Inc., Santa Clara, CA, USA).

Chromatographic separation was performed using an HP-5MS capillary column (60 m × 0.25 mm i.d., 0.25 µm film thickness). Helium was used as the carrier gas at a constant flow rate of 1 mL/min.

The GC oven temperature program was as follows: initial temperature 50 °C (0.5 min), increased at 8 °C/min to 190 °C, then at 4 °C/min to 280 °C and held for 10 min. The injector temperature was set at 280 °C with a split ratio of 50:1. The injection volume was 1 µL.

The mass spectrometer operated with an electron ionization energy of 70 eV. The ion source, quadrupole, and transfer line temperatures were maintained at 230 °C, 150 °C, and 280 °C, respectively. Mass spectra were recorded in the range of 45–450 m/z after an 8 min solvent delay. Compound identification was performed by comparison with the NIST mass spectral library (version 2.3) [21].

### 2.4. HPLC Analysis

High-performance liquid chromatography (HPLC) analysis was carried out using a Shimadzu LC-40 liquid chromatograph (Shimadzu Corporation, Kyoto, Japan). A 10 µL aliquot of the plant extract was injected into a C18 chromatographic column (250 mm × 4.6 mm, 5 µm particle size).

The mobile phase consisted of acetonitrile and 1% acetic acid in water at a flow rate of 1 mL/min. The column temperature was maintained at 40 °C. Data acquisition and processing were performed using Shimadzu LabSolutions LC/GC software (version 5.114; Shimadzu Corporation, Kyoto, Japan).

Reference standards including catechin, epicatechin, and naringin were used for compound identification based on retention times and peak area comparison [22].

### 2.5. Determination of Antioxidant Activity (FRAP Assay)

The antioxidant activity of the plant extract was determined using the ferric reducing antioxidant power (FRAP) assay. Briefly, 0.1 mL of the test solution (0.25–1.0 mg/mL) was mixed with 0.25 mL of 0.2 M phosphate buffer (pH 6.6) and 0.25 mL of 1% potassium hexacyanoferrate (III).

The mixture was incubated at 50 °C for 20 min. The reaction was terminated by adding 0.25 mL of 10% trichloroacetic acid followed by centrifugation at 3000 rpm for 10 min. The supernatant (0.5 mL) was mixed with 0.5 mL distilled water and 0.1 mL of 0.1% FeCl_3_ solution.

Absorbance was measured at 700 nm. Gallic acid was used as the reference antioxidant. All measurements were performed in triplicate [23].

### 2.6. Determination of Antiradical Activity (DPPH Assay)

Antiradical activity was evaluated using the DPPH radical scavenging assay. A 0.1 mL aliquot of extract solution (0.25–1.0 mg/mL) was mixed with 3 mL of 6 × 10^−5^ M DPPH solution.

The mixture was incubated in the dark for 30 min and absorbance was measured at 520 nm. Radical scavenging activity (ARA) was calculated according to the following equation:

ARA (%) = (A_0_ − A_t_)/A_0_ × 100

where A_0_ is the absorbance of the control and A_t_ is the absorbance of the sample.

All measurements were performed in triplicate [24].

The tested concentrations refer to the prepared extract solutions used in the assay. The IC50 value was estimated from the concentration–response data by linear interpolation between the two concentrations surrounding 50% radical scavenging activity.

### 2.7. Cytotoxicity Assay

Cytotoxic activity of the extract was evaluated using the *Artemia salina* lethality assay. *Artemia salina* eggs were incubated in artificial seawater under aeration until larvae hatched.

One-day-old larvae were exposed to different concentrations of the plant extract. The experiments were conducted in triplicate at 20 ± 2 °C under natural photoperiod conditions. The mortality of larvae in the treated groups was compared with the control group.

The extract was considered toxic if larval mortality exceeded 50% [25].

### 2.8. Ethics Statement

No ethical approval was required for this study. The toxicity assessment was conducted using *Artemia salina* nauplii as a preliminary invertebrate model; therefore, separate ethics committee approval was not required.

## 3. Results

This section presents the morphological, anatomical, phytochemical, and biological activity results obtained from the study of *Nanophyton iliense* U.P. Pratov.

### 3.1. Morphological Characteristics of Nanophyton iliense

**Figure 1:**
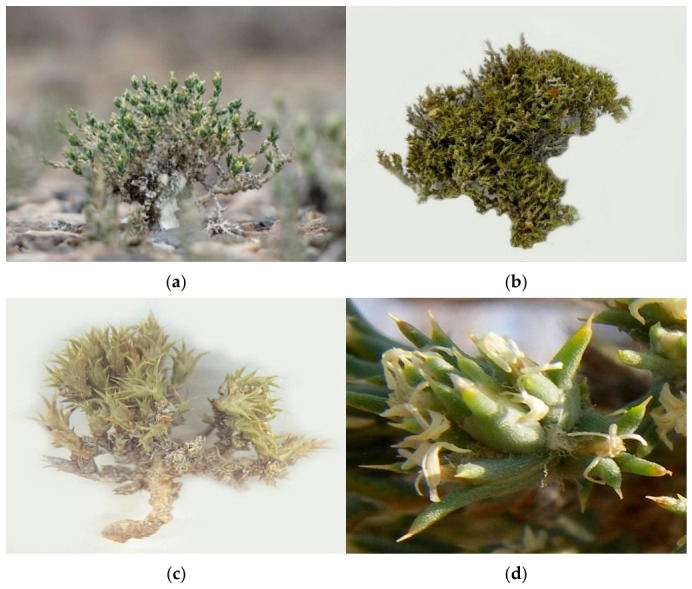
Morphological features of *Nanophyton iliense* U.P. Pratov: (**a**) plant growing in its natural habitat in the Bugaty foothills; (**b**) air-dried plant material used for analysis; (**c**) whole plant specimen showing the characteristic cushion-shaped growth habit; (**d**) close-up view of the reproductive structures of the plant.

The morphological characteristics of *N. iliense* U.P. Pratov were investigated using plant material collected from the Bugaty foothills during the flowering stage. *N. iliense* is a perennial halophytic semi-shrub characterized by a compact cushion-shaped growth habit, which represents an adaptation to arid and saline environments. The collected specimens exhibited the characteristic morphological features of the species, including densely branched shoots, succulent leaves, and reproductive structures typical of the genus. Representative images of the plant in its natural habitat, air-dried material, whole plant morphology, and reproductive organs are presented in Figure 1 and were used to confirm the botanical identity of the studied material.

Additional information on the developmental cycle, root system *morphology*, *leaf morphology*, and anatomical structure of *N. iliense* is provided in the Supplementary Materials (Figures S1–S3) [26,27,28,29].

### 3.2. Extraction Technology of Nanophyton iliense

**Figure 2:**
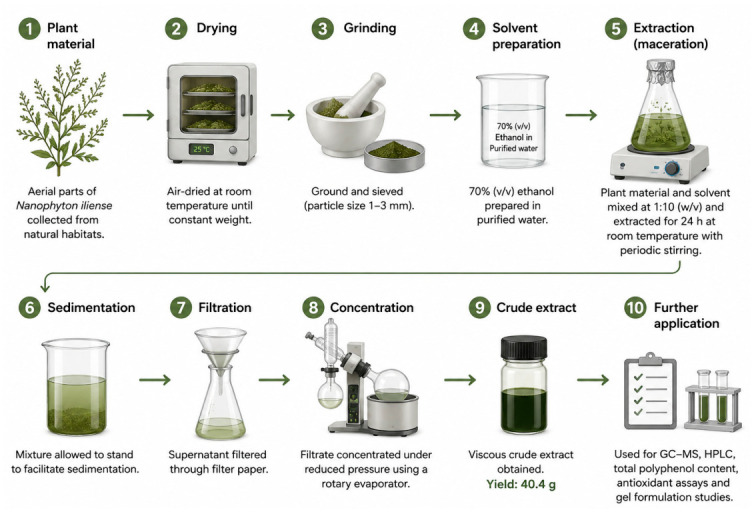
Graphical representation of the extraction process of Nanophyton iliense from plant material to crude extract.

The extraction of bioactive compounds from *N. iliense* was performed using a systematically designed process aimed at maximizing the recovery of chemically diverse constituents while preserving their stability. The extraction strategy was based on the assumption that the plant contains phytochemicals of varying polarity, which justified the use of a hydroethanolic solvent system [30]. The overall extraction workflow is presented in Figure 2.

The process included raw material preparation, hydroethanolic extraction, clarification, and concentration steps leading to the formation of a crude extract. The aerial parts of the plant were dried and ground to a particle size of 1–3 mm to enhance mass transfer efficiency. Extraction was carried out using 70% (*v*/*v*) ethanol at a plant-to-solvent ratio of 1:10 (*w*/*v*) for 24 h at room temperature with periodic stirring [30].

Following extraction, the mixture was allowed to settle and subsequently filtered to remove insoluble residues. The resulting filtrate was concentrated under reduced pressure using a rotary evaporator, yielding a viscous crude extract. The extraction process resulted in approximately 40.4 g of extract, indicating efficient recovery of bioactive compounds [30].

The obtained extract was stored at 4 °C and subsequently used for phytochemical profiling, antioxidant activity evaluation, and formulation into a gel-based delivery system [4].

### 3.3. Total Polyphenol Content

The total polyphenol content of *N. iliense* extract was determined using the Folin–Ciocalteu method and expressed as gallic acid equivalents (GAE). The assay revealed a polyphenol content of 485.05 mg GAE/L, indicating an exceptionally high concentration of phenolic compounds in the analyzed extract [31].

In comparison with other plant extracts analyzed under identical conditions, *N. iliense* demonstrated markedly higher polyphenol levels (Table 1). The content observed in this study substantially exceeded those of Calendula officinalis (24.71 mg GAE/L), Chamomilla recutita (37.39 mg GAE/L), Leonurus species (46.81 mg GAE/L), Potentilla species (78.55 mg GAE/L), and even Helichrysum arenarium (171.86 mg GAE/L), highlighting the distinctive phytochemical profile of the studied species.

Such a high accumulation of phenolic compounds is consistent with the ecological characteristics of *N. iliense* as a halophytic plant adapted to arid and saline environments [15,16,17,32]. Under such conditions, plants typically experience elevated oxidative stress, which stimulates the biosynthesis of phenolic secondary metabolites with protective functions.

Overall, these results identify *N. iliense* as an exceptionally rich source of phenolic antioxidants, supporting its potential application in pharmaceutical formulations, particularly in topical and semi-solid delivery systems [5,7].

### 3.4. GC–MS Analysis of Nanophyton iliense Extracts

GC–MS analysis of *N. iliense* extracts collected at different sampling periods (May–June) revealed a chemically diverse profile dominated by lipophilic constituents [4,22], as detailed in Supplementary Tables S1–S6. The major constituents identified across different sampling periods accounted for a substantial proportion of the total chromatographic profile. The chemical composition was predominantly represented by terpenoids, phytosterols, fatty acid derivatives, and long-chain hydrocarbons [18,19,33].

The GC–MS data are presented as relative peak area percentages; therefore, these results should be interpreted as semi-quantitative phytochemical profiling rather than absolute quantitative determination. The differences observed between sampling dates were interpreted descriptively and were not subjected to statistical significance testing.

In addition to GC–MS analysis, a comprehensive phytochemical investigation of *N. iliense* using high-resolution LC-DAD-QToF-MS has been previously reported, enabling the tentative identification of 81 metabolites [4]. These compounds included hydroxycinnamic acid amides, phenolic acids, flavonoids (including glycosides), amino acids, organic acids, sulfated derivatives, and nucleosides, reflecting a high level of chemical diversity [22].

Among the identified constituents, the flavonoid isorhamnetin-3-O-rutinoside (narcissin) was isolated and structurally confirmed, while an additional metabolite with the molecular formula C_1__7_H_1__4_O_5_ was detected but could not be conclusively characterized.

The predominance of phenylpropanoid-derived compounds observed in the LC-MS study complements the GC–MS findings and highlights the presence of both polar and non-polar bioactive constituents in the extract [4].

Despite some quantitative variability between sampling dates, the overall GC–MS profile remained consistent. Squalene was identified as the predominant compound in several samples, with relative abundances ranging from 26.69% to 96.42%, indicating its dominant contribution to the non-polar fraction. Phytol acetate was also consistently detected in high amounts (37.94–42.43%), suggesting a stable presence of diterpenoid derivatives across all analyzed samples.

Phytol-related diterpenoid derivatives were therefore considered representative components of the lipophilic fraction of the extract. However, because the GC–MS data were based on relative peak area percentages, these values should not be interpreted as absolute phytol content or purity.

Fatty acid methyl esters, including hexadecanoic acid methyl ester and unsaturated C18 derivatives (linoleic and oleic acid methyl esters), were detected in all samples, contributing to the lipid-rich composition of the extract. In addition, phytosterols such as β-sitosterol and stigmasterol, along with triterpenoids including β-amyrin, were identified, indicating the presence of structurally diverse secondary metabolites with established biological relevance.

The predominance of lipophilic compounds observed in the GC–MS profile can be explained by the extraction conditions and solvent properties, which favor the recovery of non-polar constituents [30]. Such composition is typical for plant extracts obtained under conditions promoting the diffusion of hydrophobic molecules from plant matrices.

Overall, the combined GC–MS and LC-MS data demonstrate a clear predominance of both lipophilic and phenolic bioactive compounds. From a pharmaceutical perspective, these compounds are of particular interest due to their ability to interact with biological membranes, contributing to membrane stabilization and potentially enhancing the penetration of active substances in topical delivery systems [5,8]. This aspect is especially relevant for the development of gel-based formulations.

### 3.5. HPLC Analysis of Nanophyton iliense Extract

**Figure 3:**
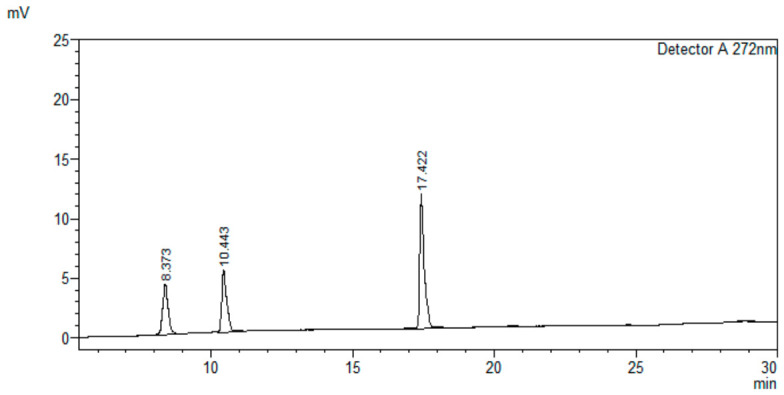
Chromatogram of reference standards detected at 272 nm.

HPLC analysis of *N. iliense* extract was performed under the chromatographic conditions described above. The chromatogram of the reference standards used for compound identification is presented in Figure 3, and the corresponding analytical parameters are summarized in Table 2.

**Figure 4:**
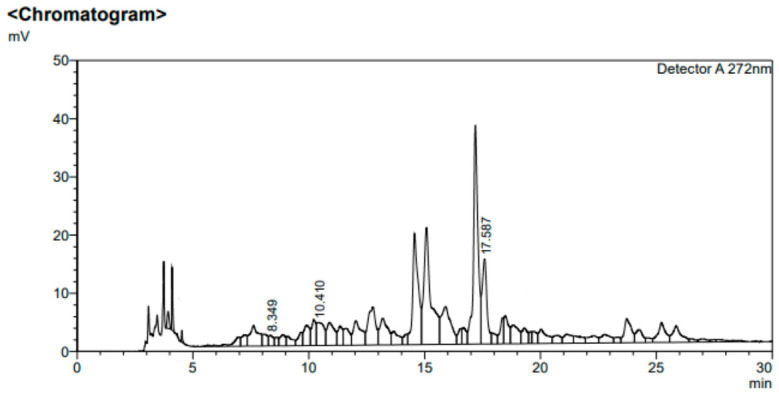
HPLC chromatogram of *Nanophyton iliense* extract detected at 272 nm.

The chromatographic analysis of the plant extract was carried out under identical conditions. The resulting chromatogram is shown in Figure 4, while the quantitative results are presented in Table 3. The chromatographic profiles of the standards and the extract demonstrated well-defined peaks at comparable retention times, indicating the presence of target phenolic compounds.

A comparison of retention times confirmed the presence of catechin, epicatechin, and naringin in the analyzed extract. The retention times of the detected peaks (8.349, 10.410, and 17.587 min) were in good agreement with those of the reference standards (8.373, 10.443, and 17.422 min), supporting reliable compound identification.

The HPLC results were used for preliminary marker identification and concentration estimation based on reference standards; therefore, they should not be interpreted as a fully validated quantitative analytical method.

Preliminary concentration estimation indicated that naringin was the predominant phenolic marker (101.732 mg/L), followed by epicatechin (79.119 mg/L) and catechin (25.639 mg/L). The predominance of naringin indicates that flavonoid glycosides play a central role in the phenolic profile of the extract. The chromatogram of the extract (Figure 4) shows that the peak corresponding to naringin exhibits the highest intensity, which is consistent with its dominant concentration in the sample.

The identified compounds belong to the flavonoid class of phenolic substances, which are known to play a key role in antioxidant defense mechanisms, particularly in plants adapted to arid and saline environments [1,2,34]. The accumulation of these compounds is consistent with the adaptive strategies of halophytic species, where secondary metabolites contribute to protection against oxidative stress [15,16,17,32].

These findings indicate that flavonoids constitute a major fraction of the phenolic profile of *N. iliense* extract. The presence of these compounds is in good agreement with the antioxidant activity observed in the FRAP and DPPH assays, supporting their contribution to the redox properties of the extract [31,35].

Furthermore, the abundance of phenolic compounds revealed by HPLC analysis is consistent with the total polyphenol content determined spectrophotometrically, confirming the high phenolic potential of the extract. The combined GC–MS and HPLC data indicate that the extract contains both lipophilic and polar bioactive compounds, which may contribute to a broad spectrum of biological activity [8,13,36]. Such a composition is particularly promising for formulation into semi-solid dosage forms, where both lipophilic and hydrophilic components can contribute to the overall efficacy [5,6,7,37].

### 3.6. The Biological Activity of Nanophyton iliense Extract

#### 3.6.1. Antioxidant Activity (FRAP Assay)

**Figure 5:**
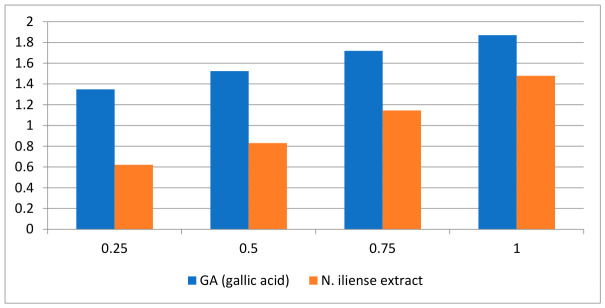
Concentration-dependent reducing activity of *N. iliense* extract compared with gallic acid in the FRAP assay.

The antioxidant activity of the *N. iliense* extract was evaluated using the ferric reducing antioxidant power (FRAP) assay, which reflects the ability of compounds present in the extract to act as electron donors and reduce Fe^3+^ to Fe^2+^ [23]. The obtained optical density values at different concentrations are presented in Table 4, while the concentration–activity relationship is illustrated in Figure 5.

The results demonstrate a clear and consistent concentration-dependent increase in reducing power. The optical density of the extract increased from 0.6211 at 0.25 mg/mL to 1.4787 at 1.0 mg/mL, indicating a progressive enhancement of antioxidant capacity with increasing concentration [23]. This behavior suggests that the active redox compounds are present in sufficient amounts and exhibit cumulative activity.

In comparison with the reference antioxidant compound gallic acid, the extract showed lower absolute values across all concentrations; however, the difference decreased significantly at higher concentrations. At 1.0 mg/mL, the extract reached approximately 79% of the activity of gallic acid (1.4787 vs. 1.8705), indicating a relatively high reducing potential.

Since the FRAP assay primarily reflects the presence of reductive compounds such as phenolics and flavonoids [1,2,23], the observed activity suggests a substantial contribution of these compounds to the overall antioxidant profile of the extract.

#### 3.6.2. Antiradical Activity

**Figure 6:**
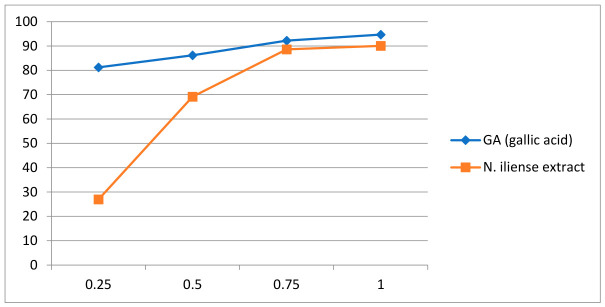
Concentration-dependent DPPH radical scavenging activity of *N. iliense* extract compared with gallic acid.

The radical scavenging activity of the extract was assessed using the DPPH assay, which measures the ability of compounds to neutralize stable free radicals [24]. The calculated antiradical activity values are presented in Table 5, and the corresponding trends are illustrated in Figure 6.

The extract exhibited a pronounced concentration-dependent increase in radical scavenging activity. At the lowest concentration (0.25 mg/mL), the activity was relatively low (26.98%), indicating limited radical neutralization under dilute conditions. However, a sharp increase was observed at 0.5 mg/mL, where the activity reached 69.18%, representing a more than twofold increase.

At higher concentrations, the extract demonstrated strong antiradical activity, reaching 88.61% at 0.75 mg/mL and 90.06% at 1.0 mg/mL. These values approach the activity of the reference compound gallic acid (94.69%), indicating that at sufficiently high concentrations, the extract exhibits comparable radical scavenging efficiency.

The steep increase in activity between 0.25 and 0.5 mg/mL suggests a threshold-like response, which is characteristic of extracts rich in phenolic compounds [1,2,24], where a critical concentration is required to effectively neutralize free radicals.

Based on the concentration–response data, the IC50 value of *N. iliense* extract in the DPPH assay was estimated by linear interpolation between 0.25 and 0.5 mg/mL, which corresponded to the two concentrations surrounding 50% radical scavenging activity. The estimated IC50 value was approximately 0.39 mg/mL under the present experimental conditions.

These results demonstrate the preliminary in vitro antioxidant potential of *N. iliense* extract. However, FRAP and DPPH assays are chemical antioxidant tests and should not be interpreted as direct evidence of topical therapeutic efficacy.

#### 3.6.3. Cytotoxicity Assessment

The cytotoxic activity of the extract was evaluated using the *Artemia salina* lethality assay as a preliminary model for biological safety [25]. The results are summarized in Table 6.

The extract of *N. iliense* did not exhibit cytotoxic effects in the *Artemia salina* assay at the tested concentrations of 1, 5, and 10 mg/mL. The survival rate of larvae remained consistently high, and no significant mortality was observed. These findings indicate the absence of general toxicity in this preliminary invertebrate model within the tested concentration range. However, the *Artemia salina* assay cannot be used as a direct substitute for dermal safety evaluation.

In contrast, the reference compound Actinomycin D demonstrated pronounced cytotoxic activity, with mortality values ranging from 63% to 96%, confirming the sensitivity and validity of the assay [25].

### 3.7. Development of Gel Formulation and Composition Justification

To translate the phytochemical and antioxidant potential of *N. iliense* extract into a pharmaceutically applicable dosage form, a semi-solid gel formulation was developed as a potential topical delivery system [5,6,7,38]. Considering that the extract contains both polar phenolic compounds (HPLC) and non-polar lipophilic constituents such as terpenoids and phytosterols (GC–MS), the formulation strategy aimed to ensure effective incorporation of chemically diverse bioactive components into a stable and homogeneous matrix [38].

The selection of excipients was guided by the mixed chemical nature of *N. iliense* extract, which contains both polar phenolic compounds and lipophilic constituents such as terpenoids, phytosterols, and fatty acid derivatives. Therefore, the formulation required a semi-solid matrix capable of incorporating chemically diverse components while maintaining homogeneity and acceptable topical properties. Lecigel was selected as the main gelling and structuring agent because it provides a stable gel network and supports the formation of a homogeneous semi-solid system. Glycerin was included as a humectant and wetting agent to improve the hydration properties and application characteristics of the gel. Tween 80 was used as a non-ionic surfactant to facilitate the dispersion of lipophilic constituents within the aqueous gel phase and to improve the uniform distribution of the extract. Benzyl alcohol was included as a preservative to support microbiological quality during storage. Purified water served as the continuous phase for the hydrophilic fraction of the formulation.

Among the tested compositions, Gel No. 4 provided the most balanced combination of homogeneity, consistency, spreadability, and absence of greasy residue, which justified its selection for further quality evaluation. This screening was used as an initial formulation selection step and was not intended to replace detailed pharmaceutical quality testing.

A series of five experimental gel formulations were prepared using different combinations of gelling agents, surfactants, and stabilizing components. The formulations were evaluated based on key physicochemical and organoleptic parameters [39], including homogeneity, consistency, appearance, spreadability, and residual greasiness (Table 7).

The comparative analysis revealed pronounced differences in structural organization and application properties among the tested formulations. Gel No. 1 demonstrated acceptable homogeneity but was characterized by excessive fluidity and the presence of a greasy residue, which may negatively affect patient acceptability. Gel No. 2 and Gel No. 3 showed insufficient structural stability and poor homogeneity, including the presence of dispersed phases and air inclusions, indicating incomplete integration of the extract into the gel matrix. Gel No. 5, although relatively homogeneous, exhibited low viscosity and limited spreadability, which restrict its practical application.

In contrast, Gel No. 4 exhibited the most favorable characteristics among the tested samples. This formulation demonstrated a homogeneous and stable structure, optimal consistency, good spreadability, and absence of greasy residue. Such properties are essential for topical formulations, as they ensure uniform application and adequate interaction with the skin surface [5,6,7,38].

Based on these results, Gel No. 4 was selected as the optimal formulation for further development. The composition of the selected formulation is presented in Table 8.

The optimized formulation consisted of *N. iliense* extract as the active component, Lecigel as the gelling agent, glycerin as a humectant, Tween 80 as a surfactant and solubilizing agent, benzyl alcohol as a preservative, and purified water as the dispersion medium. Overall, this composition was selected as the most balanced system for further quality evaluation because it combined acceptable homogeneity, consistency, spreadability, and absence of greasy residue [39].

### 3.8. Gel Preparation Technology

**Figure 7:**
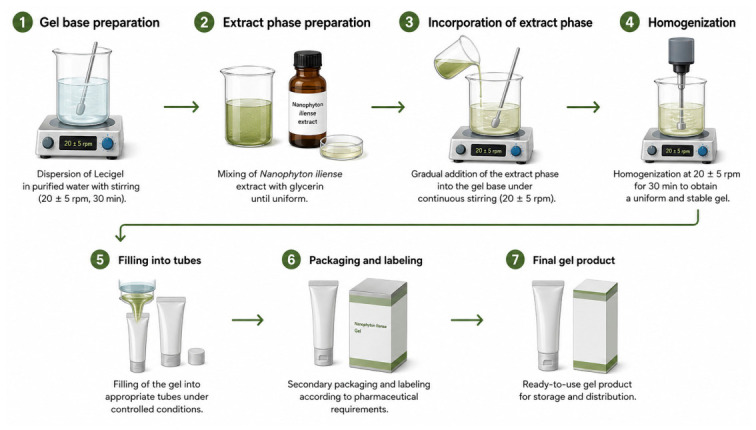
Technological stages in the preparation of *Nanophyton iliense* gel, including base preparation, extract incorporation, homogenization, and packaging.

The gel containing *Nanophyton iliense* extract was prepared using a sequential process designed to ensure uniform distribution of the active component and formation of a stable semi-solid system [40]. The overall workflow of the preparation process is presented in Figure 7.

The process began with accurate weighing of all components according to the optimized formulation. Precise measurement of each component ensured consistency and reproducibility of the formulation [40].

The gel base was prepared by dispersing Lecigel in purified water under controlled stirring conditions. Mixing was carried out at a stirring speed of 20 ± 5 rpm for approximately 30 min to ensure complete hydration of the polymer and formation of a uniform gel structure [40,41].

Separately, the extract-containing phase was prepared by mixing *N. iliense* extract with glycerin until a homogeneous system was obtained. This step facilitated uniform dispersion of the extract and improved its compatibility with the hydrophilic gel matrix [41].

The prepared extract phase was then gradually incorporated into the gel base under continuous stirring. Controlled addition prevented the formation of aggregates and ensured uniform distribution of the active component throughout the system.

Following incorporation, the formulation was subjected to homogenization at 20 ± 5 rpm for 30 min. This step was critical for obtaining a stable and homogeneous gel with consistent structural properties [40,42].

After homogenization, the gel was filled into tubes as primary packaging under controlled conditions. Secondary packaging and labeling were performed to ensure product integrity, identification, and compliance with pharmaceutical requirements [43].

Overall, the described process ensures the formation of a homogeneous gel system with uniform distribution of the active component and suitable characteristics for topical application [5,6,7,40].

### 3.9. Quality Evaluation of the Gel Formulation

The quality of the developed *Nanophyton iliense* gel was evaluated in accordance with pharmacopoeial requirements for semi-solid dosage forms, including the State Pharmacopoeia of the Republic of Kazakhstan and the Eurasian Economic Union Pharmacopoeia [20,44]. The assessment included organoleptic characteristics, identification of the active component, pH determination, homogeneity, viscosity assessment, microbiological purity, quantitative analysis, as well as packaging, labeling, and storage conditions.

The quality parameters and acceptance criteria of the developed gel are summarized in Table 9.

Identification of the active component was performed using gas chromatography in accordance with SP RK Vol. 1, Section 2.2.28, where phytol was selected as a marker compound [20,21]. The observed retention time of approximately 20.3 min confirmed the presence of the phytol marker in the gel formulation and supported the incorporation of the lipophilic fraction of *N. iliense* extract into the gel matrix.

Quantitative determination of phytol was carried out using gas chromatography in accordance with SP RK Vol. 1, Section 2.2.28 [20,21]. The phytol content was not less than 3%, supporting preliminary standardization of the formulation.

The pH of the gel was determined potentiometrically according to Ph. Eur. 2.2.3 and EAEU Pharmacopoeia requirements (Section 2.1.2.3). The formulation complied with the established acceptance criterion of pH 6.5–7.5, which is suitable for topical application and helps minimize the risk of skin irritation while maintaining formulation stability [44,45].

Homogeneity was assessed by visual examination in accordance with EAEU Pharmacopoeia (Section 2.1.9.10), confirming the physical uniformity of the gel matrix and the absence of visible particles or phase separation [44].

Microbiological purity was evaluated in accordance with pharmacopoeial methods for non-sterile dosage forms (Category 2), including total aerobic microbial count (TAMC) and total yeast and mold count (TYMC), according to SP RK Vol. 1, Sections 2.3.12, 2.6.13 and 5.1.4. The formulation complied with acceptable limits and showed an absence of pathogenic microorganisms [20,47].

Packaging, labeling, and storage conditions were established in accordance with regulatory and pharmacopoeial requirements. The gel was packaged in aluminum tubes (100 g) and stored at temperatures not exceeding 25 °C in a dry, light-protected environment to maintain its physicochemical stability [20,46,48].

Overall, the developed *N. iliense* gel meets the basic pharmacopoeial requirements evaluated in this study and is suitable for further pharmaceutical development and more detailed characterization [5,6,7,20,44].

## 4. Discussion

The present study extends previous phytochemical investigations of *Nanophyton iliense* by evaluating whether its chemically complex extract can be incorporated into a semi-solid topical formulation. While earlier work mainly focused on the identification of bioactive constituents, the present study combines phytochemical profiling, antioxidant evaluation, preliminary safety screening, and initial gel development. This approach allows the pharmaceutical relevance of *N. iliense* extract to be assessed not only as a source of bioactive compounds, but also as a candidate material for further topical formulation studies [5,6,7,12,49].

GC–MS analysis revealed a predominance of lipophilic constituents, particularly terpenoids and phytosterols, with squalene and phytol derivatives representing major components [18,19]. Such a profile is typical for halophytic species and reflects adaptive metabolic responses to environmental stress [15,16,17,32]. From a pharmaceutical standpoint, these compounds are relevant because lipophilic constituents can interact with lipid membrane structures and may contribute to dermal delivery behavior [12,50,51,52]. However, this assumption requires confirmation in future release and permeation studies.

In parallel, HPLC analysis confirmed the presence of flavonoid compounds, including catechin, epicatechin, and naringin, which are well-established antioxidants [10,34]. The coexistence of these polar phenolics with non-polar constituents highlights the amphiphilic nature of the extract. This dual composition is particularly advantageous for topical formulations, as it allows simultaneous interaction with both aqueous and lipid domains of the skin barrier [5,6,7,12,50,51].

The high total polyphenol content supports the hypothesis that *Nanophyton iliense* is a rich source of antioxidant compounds. This observation is consistent with the ecological characteristics of halophytes, which are known to accumulate phenolic metabolites as a defense mechanism against oxidative stress [15,16,17,32]. The elevated polyphenol levels are consistent with the antioxidant activity observed in FRAP and DPPH assays, where the extract exhibited a clear concentration-dependent response [31,35].

Importantly, the antioxidant activity cannot be attributed solely to phenolic compounds. The presence of lipophilic terpenoids and sterols suggests additional mechanisms, including membrane stabilization and indirect modulation of oxidative processes [18,33,49]. Therefore, the biological activity of the extract is likely governed by synergistic interactions between different classes of compounds rather than a single dominant component [21,49].

The absence of cytotoxicity in the *Artemia salina* assay indicates a favorable preliminary safety profile, which is essential for further pharmaceutical development [25]. Although this model provides only an initial assessment, the results support further evaluation of the extract in topical formulation studies.

The formulation strategy was designed to address the key challenge associated with such complex extracts, namely the incorporation of chemically diverse constituents into a stable delivery system [38,40]. The selected gel matrix, based on Lecigel, enabled the formation of a structured network capable of accommodating both hydrophilic and lipophilic components [38,40,41]. The inclusion of Tween 80 was critical for solubilizing non-polar compounds and ensuring their uniform distribution within the aqueous gel phase [13,38,41].

This formulation approach is consistent with current trends in topical drug delivery, where multifunctional excipient systems are used to improve the incorporation of poorly soluble bioactive compounds into semi-solid matrices [5,6,7,13,51]. In the present study, the selected excipient system supported the preparation of a homogeneous gel containing both hydrophilic and lipophilic constituents of *N. iliense* extract.

The performed quality evaluation confirmed several basic pharmacopoeial and technological characteristics of the optimized formulation, including acceptable appearance, homogeneity, pH compliance, viscosity assessment, marker identification, marker assay, microbiological purity, packaging, labeling, and storage conditions [20,44,45,46,47,48]. These results support the selection of Gel No. 4 for further pharmaceutical development.

Phytol was used as a technological marker compound because it represents the lipophilic fraction of *N. iliense* extract detected by GC–MS and allows confirmation of the incorporation of this fraction into the gel matrix [20,21]. However, the extract is chemically complex and contains both lipophilic constituents and polar phenolic compounds, including catechin, epicatechin, and naringin [10,18,19,34].

Therefore, phytol alone cannot fully represent the entire phytochemical profile of the extract. Future standardization of the gel should include a broader marker system combining a lipophilic marker, such as phytol, with phenolic markers such as naringin, epicatechin, or catechin. In addition, content uniformity should be evaluated by analyzing samples collected from different parts of the gel batch to confirm the homogeneous distribution of marker compounds throughout the formulation.

The present study should be regarded as a preliminary formulation and quality evaluation rather than a complete pharmaceutical characterization. The antioxidant activity was assessed using in vitro chemical assays, which do not fully reflect the complexity of biological skin systems [23,24].

Similarly, the *Artemia salina* assay provided only an initial general toxicity screen and cannot replace dermal irritation, sensitization, or mammalian skin cell cytotoxicity testing [25]. Detailed rheological profiling, quantitative spreadability testing with units, extrudability assessment, syneresis evaluation, long-term stability studies, and release/permeation testing should therefore be included in further pharmaceutical development [11,12,50,51,52].

In addition, full analytical validation of the GC–MS and HPLC methods, including linearity, precision, accuracy, LOD/LOQ, repeatability, and sample preparation reproducibility, should be included in future standardization studies of the extract and gel formulation.

Taken together, the obtained results show that *N. iliense* extract combines phenolic and lipophilic constituents with marked antioxidant activity and a favorable preliminary safety profile. Its successful incorporation into a homogeneous gel formulation supports further investigation of this system for topical pharmaceutical application [5,6,7,51,52].

## 5. Conclusions

This study showed that *Nanophyton iliense* extract contains both lipophilic and phenolic bioactive constituents and demonstrates marked antioxidant activity in FRAP and DPPH assays. The absence of cytotoxicity in the *Artemia salina* model supports its preliminary safety within the tested concentration range.

The extract was successfully incorporated into a Lecigel-based semi-solid formulation. Among the tested samples, Gel No. 4 demonstrated the most suitable characteristics, including homogeneity, appropriate consistency, good spreadability, and absence of greasy residue.

These results support the potential of *N. iliense* as a source of antioxidant compounds for topical formulation development. Further studies should focus on release behavior, skin permeation, long-term stability, and in vivo or advanced ex vivo evaluation.

## Figures and Tables

**Table 1:** Total polyphenol content of selected plant extracts determined under identical Folin–Ciocalteu assay conditions.

Sample	Total Polyphenols (mg GAE/L)
*Nanophyton iliense*	485.05
*Calendula officinalis*	24.71
*Chamomilla recutita*	37.39
*Leonurus* sp.	46.81
*Helichrysum arenarium*	171.86
*Potentilla* sp.	78.55

**Table 2:** Results of HPLC analysis of reference standards.

Retention Time (min)	Area	Height	Concentration (mg/L)	Name
8.373	63,691	4214	64.828	Catechin
10.443	71,059	5162	64.272	Epicatechin
17.422	132,983	11,244	64.313	Naringin

**Table 3:** Results of HPLC analysis of the extract of *N. iliense* U.P. Pratov.

Retention Time (min)	Area	Height	Concentration (mg/L)	Name
8.349	25,189	1816	25.639	Catechin
10.410	87,473	3972	79.119	Epicatechin
17.587	210,358	14,597	101.732	Naringin

**Table 4:** FRAP absorbance values of *N. iliense* extract and gallic acid at different concentrations.

No.	Samples	Optical Density Value at Concentration (mg/mL)			
		0.25	0.5	0.75	1.0
1	Gallic acid (GA)	1.3480	1.5237	1.7191	1.8705
2	Extract of *Nanophyton iliense* U.P. Pratov (*N. iliense*)	0.6211	0.8301	1.1445	1.4787

**Table 5:** DPPH radical scavenging activity (%) of *N. iliense* extract and gallic acid at different concentrations.

No.	Samples	Optical Density Value at Concentration (mg/mL)			
		0.25	0.5	0.75	1.0
1	Gallic acid (GA)	81.22	86.19	92.21	94.69
2	Extract of *Nanophyton iliense* U.P. Pratov (*N. iliense*)	26.98	69.18	88.61	90.06

**Table 6:** Results of cytotoxic activity evaluation.

Studied Substances	Concentration (mg/mL)	Number of Larvae in Control		Number of Larvae in Sample			Survival of Larvae in Control (%)	Survival of Larvae in Sample (%)	Mortality, A (%)	Neurotoxicity (%)
		Alive	Dead	Alive	Dead	Paralyzed				
Actinomycin D	10	22	0	0	22	0	96	0	96	0
	5	22	0	1	25	0	96	4	92	0
	1	22	0	9	18	0	96	33	63	0
Extract of *Nanophyton iliense* U.P. Pratov (*N. iliense*)	10	22	0	25	1	0	96	96	0	0
	5	22	0	25	1	0	96	96	0	0
	1	22	0	26	1	0	96	96	0	0

**Table 7:** Preliminary organoleptic and technological screening of experimental gel formulations.

Formulation	Homogeneity	Consistency	Appearance	Spreadability	Residue	Decision
Gel 1	Homogeneous	Fluid	Yellowish	Moderate	Greasy	Rejected
Gel 2	Non-homogeneous	Thick	Opaque	Poor	Non-greasy	Rejected
Gel 3	Non-homogeneous	Very thick	Yellow	Low	Non-greasy	Rejected
Gel 4	Homogeneous	Soft	Transparent yellow	Good	Non-greasy	Selected
Gel 5	Homogeneous	Very fluid	Yellowish	Low	Non-greasy	Rejected

**Table 8:** Composition and functional role of excipients in the optimized gel formulation.

Component	Amount (g)	Function
*Nanophyton iliense* extract	3.0	Active component
Lecigel	3.0	Gelling agent
Glycerin	8.0	Humectant
Tween 80	2.5	Emulsifier/solubilizer
Benzyl alcohol	0.5	Preservative
Purified water	up to 100	Vehicle

**Table 9:** Quality evaluation parameters of the optimized *Nanophyton iliense* gel formulation.

Parameter	Specification	Method/Reference
Description	Light-yellow homogeneous gel with characteristic odor	Visual (organoleptic) evaluation, SP RK Vol. 1, Sections 2.2.1 and 2.2.2 [20]
Identification	Phytol detected; retention time approximately 20.3 min; identification probability 92%	Gas chromatography, SP RK Vol. 1, Section 2.2.28 [20,21]
pH	Complies with the acceptance criterion of 6.5–7.5	Potentiometric method, Ph. Eur. 2.2.3/EAEU Pharmacopeia 2.1.2.3 [44,45]
Homogeneity	Uniform, no visible particles or phase separation	Visual examination, EAEU Pharmacopeia 2.1.9.10 [44]
Viscosity	Evaluated by capillary viscometry	Pharmacopoeial method/technological quality assessment [20,44]
Assay (phytol)	Not less than 3%	Gas chromatography (quantitative), SP RK Vol. 1, Section 2.2.28 [20,21]
Packaging	Aluminum tubes (100 g)	Regulatory requirements [46]
Labeling	Complete product information	Regulatory requirements [46]
Microbiological purity	Complies with Category 2 limits; absence of pathogens	TAMC and TYMC for non-sterile dosage forms, SP RK Vol. 1, Sections 2.3.12, 2.6.13 and 5.1.4 [20,47]
Storage	≤25 °C, protected from light and moisture	SP RK and regulatory storage/GMP requirements [20,46,48]

## Data Availability

The original contributions presented in this study are included in the article/Supplementary Material. Further inquiries can be directed to the corresponding authors.

## Supplementary Materials

The following supporting information can be downloaded at: https://www.mdpi.com/article/10.3390/pharmaceutics18060710/s1. Supplementary Section S1: Additional morphological and anatomical characteristics of *Nanophyton iliense*; Figure S1: Root system morphology of *Nanophyton iliense*; Figure S2: Leaf morphology of *Nanophyton iliense*; Figure S3: Microscopic structure of the leaf of *Nanophyton iliense*; Tables S1–S6: GC–MS identified compounds in *Nanophyton iliense* extracts obtained from different sampling dates (May–June).

## Abbreviations

The following abbreviations are used in this manuscript:

LC-MS	Liquid Chromatography–Mass Spectrometry
HPLC	High-Performance Liquid Chromatography
FRAP	Ferric Reducing Antioxidant Power
DPPH	2,2-Diphenyl-1-picrylhydrazyl
ARA	Antiradical Activity
GA	Gallic Acid
*N. iliense*	*Nanophyton iliense*
NIST	National Institute of Standards and Technology
IPNI	International Plant Names Index
POWO	Plants of the World Online
