# Supplementary material for: Development and Characterization of a Nanophyton iliense-Based Gel for Topical Application
Source: Pharmaceutics. 2026 Jun 9;18(6):710. doi: 10.3390/pharmaceutics18060710 (PMC13306694; doi:10.3390/pharmaceutics18060710)
Supplement: Supplementary file 1 [file pharmaceutics-18-00710-s001.zip › pharmaceutics-4344249-supplementary.pdf]

Additional Morphological and Anatomical Characteristics of *Nanophyton iliense*

Supplementary Section S1. Additional morphological and anatomical characteristics of *Nanophyton iliense*

The detailed developmental cycle, root system morphology, leaf morphology, and leaf anatomy of *N. iliense* are presented below. These data provide additional botanical characterization of the plant material used in the study.

S1.1. Developmental cycle and shoot morphology

During the first year of vegetation, *N. iliense* grows monopodially and does not branch. The main vegetative growth period ends by June, after which plants enter a period of summer dormancy. By the end of the growing season, individuals remain at the juvenile stage, reaching a height of 2–4 cm and forming 15–20 metameres [26,27].

Large vegetative buds develop in the middle part of the first-order shoot and give rise to second-order shoots during the following growing season [27].

During the second year of vegetation, monopodial growth of the first-order shoot continues but remains insignificant (1–2 cm). Second-order shoots grow to a length of 2–3 cm, indicating the transition of the plants to the immature developmental stage [26].

During the third year of vegetation, the main axis (first-order shoot) may remain present or partially die off. At this stage, some second-order shoots begin flowering and fruiting [20].

By the fourth year of vegetation, monopodial growth ceases and the apex of the first-order shoot dies. Plants completely transition to sympodial growth [27].

Annual shoots develop from axillary buds of the residual shoot. Renewal buds begin forming in mid-April as the shoot develops. During the first ten days of May, four primordia of bud scales form with a plastochron of 5–6 days. By the end of May, the number of bud scales reaches seven, after which intrabud organogenesis ceases.

By late autumn, buds reach 2–5 mm in height and approximately 2 mm in width. The buds are classified as semi-open [27].

Annual shoots of *N. iliense* are orthotropic and occur in four morphological types [20]:

- Very short vegetative shoots;
- Longer renewal shoots;
- Specialized generative shoots;
- Non-specialized generative shoots.

Vegetative shoots develop from buds formed during the previous year or arise as a continuation of monopodial growth [20].

Generative shoots develop over a 3–4 year cycle. During the first year, a bud forms; during the second year, a vegetative shoot develops; and during the third year, monopodial growth continues and an inflorescence is formed [27].

Annual growth typically reaches 10–15 mm, while the total length of a non-specialized generative shoot is 2–3 cm. Similar to saxaul, the plant may continue to grow a terminal vegetative shoot after specialized generative shoots have formed at its base.

Mature perennial plants in the Bugaty foothills reach 7–8 cm in height, with a crown diameter of 7–12–15 cm and a root collar diameter of 1–2 cm.

The number of main skeletal axes ranges from 3 to 7, each producing 3–5 secondary skeletal axes. These axes generate approximately 145–205 non-specialized generative shoots and 25–30 shoots with underdeveloped fruits.

Plants do not undergo particulation; however, at a depth of 4–5 cm from the soil surface, the main root may disintegrate and be replaced by second-order lateral roots that penetrate to depths of 120–170 cm [27].

S1.2. Root system morphology

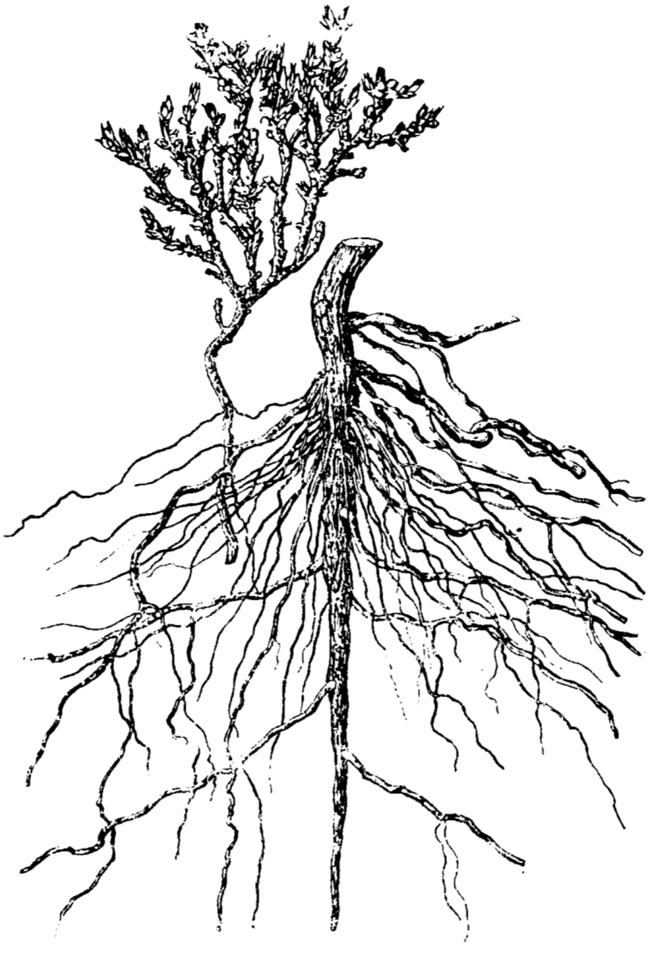

Figure S1. Root system morphology of *Nanophyton iliense* showing the taproot structure and lateral branching zones.

The root structure demonstrates significant variability depending on edaphic conditions [28].

In the Bugaty foothills, the plant develops a well-formed taproot, with the first branching occurring at a depth of approximately 10 cm. Lateral roots spread horizontally within the upper soil layer, forming the first feeding zone characterized by relatively high alkalinity [28].

At a depth of approximately 42 cm, the root system branches again, forming a second feeding zone located within gravelly and relatively moist soil horizons. The species demonstrates tolerance to weak chloride salinity and exhibits a high level of resistance to alkaline soils [28].

Based on the observed morphological features, including prolonged monopodial shoot growth, low annual increment, and suppressed vertical growth, *N. iliense* can be classified as a dwarf shrub life form [26,28].

S1.3. Leaf morphology

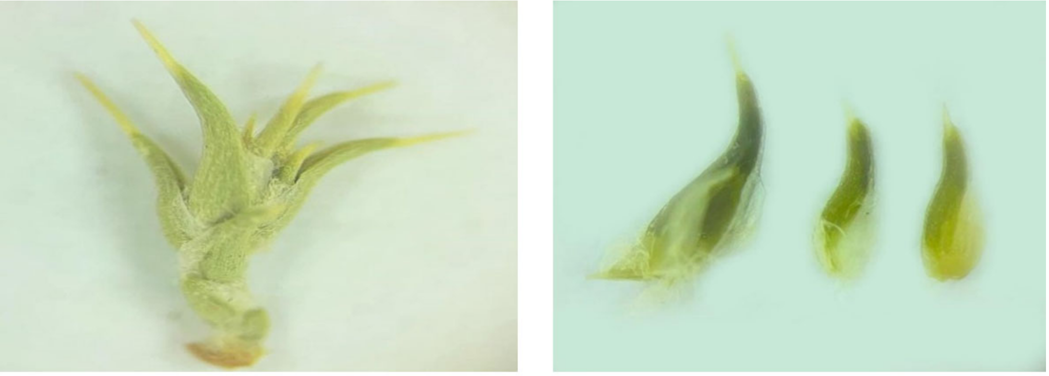

(a) (b)

Figure S2. Leaf morphology of *Nanophyton iliense* U.P. Pratov: (a) sessile leaf attached to the shoot; (b) isolated leaves demonstrating the characteristic sickle-shaped morphology.

Leaves are small, 2.5–4.0 mm long, sessile, and composed of four structural zones.

Approximately two-thirds of the leaf is occupied by an expanded sickle-shaped sheath with membranous margins that partially encloses the shoot [29].

S1.4. Leaf anatomy

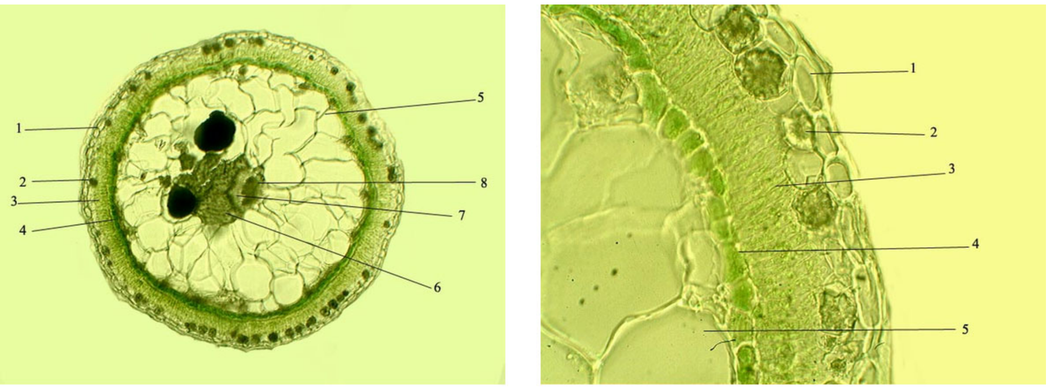

(a) (b)

Figure S3. Microscopic structure of the leaf of *Nanophyton iliense*: 1-epidermis; 2-parenchyma with calcium oxalate druses; 3-palisade parenchyma; 4-chlorenchyma; 5-water-storing tissue; 6-sclerenchyma strands; 7-xylem; 8-phloem.

The mesophyll exhibits a Kranz-type ventrodorsal structure. Beneath the abaxial epidermis lies a single row of palisade parenchyma followed by radially elongated Kranz sheath cells associated with lateral vascular bundles and aquiferous parenchyma.

The main vein contains a small number of vascular elements consisting of small vessels and phloem [29].

A prominent sclerenchyma strand occupies a large portion of the leaf cross-section.

Aquiferous parenchyma is relatively sparse. Its cells are thick-walled and located adjacent to the epidermis on the adaxial side of the leaf. Cells of the aquiferous parenchyma in contact with the sclerenchyma strand contain large calcium oxalate druses.

Toward the center of the leaf, the cross-section remains crescent-shaped; however, chlorenchyma becomes distributed on both sides of the leaf.

The leaf itself is extremely short (0.2–0.3 mm) and possesses a cranzocentric scleromorphic mesophyll type lacking hypodermis. The central region contains a thick sclerenchymatous sheath surrounded by a nearly continuous layer of idioblasts containing calcium oxalate druses. The leaf apex terminates in a spine.

Leaf sclerenchymatization begins very early during development. Sclerenchyma tissues differentiate from the procambium simultaneously with vascular tissue differentiation when the leaf reaches 150–200 μm in length.

The epidermis is composed of small cells with straight thickened walls. Stomata are arranged in rows on both surfaces of the leaf and belong to hemiparacytic and paracytic types. Numerous paired stomata are present.

Leaves remain on the shoots until the end of the growing season and do not abscise [29].

Note: Reference numbers correspond to the reference list of the main manuscript.
